# The flattening of spacetime hierarchy of the *N,N*-dimethyltryptamine brain state is characterized by harmonic decomposition of spacetime (HADES) framework

**DOI:** 10.1093/nsr/nwae124

**Published:** 2024-04-04

**Authors:** Jakub Vohryzek, Joana Cabral, Christopher Timmermann, Selen Atasoy, Leor Roseman, David J Nutt, Robin L Carhart-Harris, Gustavo Deco, Morten L Kringelbach

**Affiliations:** Centre for Eudaimonia and Human Flourishing, Linacre College, Department of Psychiatry, University of Oxford, Oxford OX3 9BX, UK; Department of Psychiatry, University of Oxford, Oxford OX3 7JX, UK; Center for Music in the Brain, Aarhus University, Aarhus 8000, Denmark; Center for Brain and Cognition, Computational Neuroscience Group, Department of Information and Communication Technologies, Universitat Pompeu Fabra, Barcelona 08005, Spain; Centre for Eudaimonia and Human Flourishing, Linacre College, Department of Psychiatry, University of Oxford, Oxford OX3 9BX, UK; Life and Health Sciences Research Institute, School of Medicine, University of Minho, Braga 4710-057, Portugal; ICVS/3B's - PT Government Associate Laboratory, Braga/Guimarães 4710-057, Portugal; Centre for Psychedelic Research, Department of Brain Sciences, Imperial College London, London SW7 2AZ, UK; Centre for Eudaimonia and Human Flourishing, Linacre College, Department of Psychiatry, University of Oxford, Oxford OX3 9BX, UK; Department of Psychiatry, University of Oxford, Oxford OX3 7JX, UK; Centre for Psychedelic Research, Department of Brain Sciences, Imperial College London, London SW7 2AZ, UK; Centre for Psychedelic Research, Department of Brain Sciences, Imperial College London, London SW7 2AZ, UK; Centre for Psychedelic Research, Department of Brain Sciences, Imperial College London, London SW7 2AZ, UK; Departments of Neurology and Psychiatry, University of California San Francisco, San Francisco 94143, USA; Center for Brain and Cognition, Computational Neuroscience Group, Department of Information and Communication Technologies, Universitat Pompeu Fabra, Barcelona 08005, Spain; Institució Catalana de la Recerca i Estudis Avançats (ICREA), Barcelona 08010, Spain; Department of Neuropsychology, Max Planck Institute for Human Cognitive and Brain Sciences, Leipzig, Germany; School of Psychological Sciences, Monash University, Melbourne, Australia; Centre for Eudaimonia and Human Flourishing, Linacre College, Department of Psychiatry, University of Oxford, Oxford OX3 9BX, UK; Department of Psychiatry, University of Oxford, Oxford OX3 7JX, UK; Center for Music in the Brain, Aarhus University, Aarhus 8000, Denmark

**Keywords:** spatio-temporal brain dynamics, DMT, harmonic modes

## Abstract

The human brain is a complex system, whose activity exhibits flexible and continuous reorganization across space and time. The decomposition of whole-brain recordings into harmonic modes has revealed a repertoire of gradient-like activity patterns associated with distinct brain functions. However, the way these activity patterns are expressed over time with their changes in various brain states remains unclear. Here, we investigate healthy participants taking the serotonergic psychedelic *N,N*-dimethyltryptamine (DMT) with the Harmonic Decomposition of Spacetime (HADES) framework that can characterize how different harmonic modes defined in space are expressed over time. HADES demonstrates significant decreases in contributions across most low-frequency harmonic modes in the DMT-induced brain state. When normalizing the contributions by condition (DMT and non-DMT), we detect a decrease specifically in the second functional harmonic, which represents the uni- to transmodal functional hierarchy of the brain, supporting the leading hypothesis that functional hierarchy is changed in psychedelics. Moreover, HADES’ dynamic spacetime measures of fractional occupancy, life time and latent space provide a precise description of the significant changes of the spacetime hierarchical organization of brain activity in the psychedelic state.

## Teaser

To understand the effects of the classical serotonergic psychedelic N,N-Dimethyltryptamine (DMT), researchers have employed the novel HADES framework revealing changes to functional spacetime hierarchy under DMT.

## INTRODUCTION

The brain is endowed with complex dynamics and can be perceived along spatial and temporal dimensions [1]. Traditionally, neuroscience has focused on delineating and studying localized cortical regions to map brain function in a temporarily static fashion [2]. However, recent developments in neuroscience have started to indicate more spatially continuous representations of functional topography [3,4], and at the same time to stress the importance of temporally varying brain dynamics [5]. Indeed, the notion of brain spacetime has been described as a ‘common currency’ between the neuronal and mental brain features [6,7], and has been used to delineate various altered states of consciousness [8]. Despite such progress, it remains unknown what underlying mechanisms drive, on one hand, the gradient-like organization of cortical topography, and on the other, the waning and waxing of the brain's spatiotemporal patterns of activity.

Here we investigate one of the most potent psychedelic (i.e. ‘mind-manifesting’) experiences induced by the *N,N*-dimethyltryptamine (DMT)—a naturally occurring serotonergic psychedelic [9]. Unlike psilocybin and lysergic acid diethylamide, its expression is marked by a short duration of the psychedelic experience. It is often associated with alterations in visual and somatic effects. At high doses, a complete dissociation from the external environment precedes an immersion into mental worlds or dimensions described as ‘other’ but not less ‘real’ than the one inhabited in normal waking consciousness. Such experiences correlate with subjective rating items such as ‘I experienced a different reality or dimension’, ‘I saw geometric patterns’ and ‘I felt unusual bodily sensations’ [10,11]. It is these qualities of one's conscious experience that motivate a renewed interest in DMT drawing parallels with phenomena such as the near-death experience (NDE) and dreaming [12].

Furthermore, like other psychedelics, DMT may have clinical relevance and is currently being trialled for the treatment of depressive symptoms [13,14]. Studies with Ayahuasca, containing DMT itself as well as monoamine oxidase inhibitors (MAOIs), have shown promising results in patients with depression [15]. However, further investigations exploring the neural and plasticity dynamics of DMT experiences are necessary to provide mechanistic accounts for the relevance of DMT and related psychedelics for the treatment of mental health disorders [16–18].

In the brain, psychedelics enhance the richness of spatiotemporal dynamics along both the temporal and spatial dimensions. This has been corroborated by repertoire broadening of functional states and increases in temporal complexity as well as shifting of the brain to a more integrated state with the subversion of functional systems [19–22]. Consistently, neuroimaging DMT has revealed an increase in global functional connectivity—featuring a functional network disintegration and desegregation that is a reliable feature of the psychedelic state, and a collapse of the unimodal to transmodal functional gradient [11]. Taken together, the current findings and subjective reports are in line with the entropic [23,24] and anarchic brain [25] models, where an increase in entropy of spontaneous brain activity parallels the undermining of hierarchically organized brain function [23–25].

Here, we studied DMT with the Harmonic Decomposition of Spacetime (HADES) framework, which can describe the brain's hierarchical processing across both spatial and temporal dimensions. Historically, Brodmann's interactive atlas of cellular morphology and organization has given rise to the view of functional specialization of individual brain areas [26,27]. Spatially, this suggests a sharp delineation between cortical areas in terms of their anatomy and function. However, supported by evolutionary and developmental neuroscience [28,29], cortical gradients have challenged this view by suggesting gradually varying boundaries between and within brain regions, both in terms of function and anatomy [3,4,30]. Functionally, gradient-like organization proposes an intrinsic coordinate system of human brain organization continuously varying from unimodal to transmodal cortical areas [3,31]. Similarly, topographical maps of retinotopy, somatotopy and tonotopy have shown smooth variation of anatomy and function within brain areas [32–35].

Along the temporal dimension, studies of dynamic functional connectivity in functional magnetic resonance imaging (fMRI) have revealed the importance of characterizing the temporal features of brain activity as opposed to the static picture described by known resting-state networks [5,36]. Such approaches describe temporal functional connectivity in terms of sliding-window analysis [37], by considering the most salient events in the timeseries [38,39] constrained by structural connectivity [40,41], as a temporal process of hidden states [42,43] or as a temporal trajectory in a landscape of attractors [44,45]. Broadly, these approaches share the description of complex brain dynamics in terms of spatial patterns expressed in time and therefore can be represented in terms of the patterns’ fractional occupancy, lifetimes or probability of transitions. Uniquely, in this paper, HADES brings this dynamic perspective to the functional gradients and their temporal expression.

Importantly, HADES characterizes the brain's spatiotemporal activity in an atlas-free manner in terms of functional gradients (functional harmonics) defined in space and expressed over time. To that end, we derived functional harmonics (FHs) [4] and their temporal expression by decomposing fMRI data into FHs via harmonic decomposition [46]. The motivation for HADES is to reproduce the spatially distributed multiscale nature of functional gradients while accounting for their temporal evolution, and therefore focus the analysis on the functional gradients over time. In practice, HADES, as the decomposition of space and time, can be extended to any modality to obtain the spatial configuration of the modes over time. In this paper, the analysis of FHs renders HADES a unimodal application, which distinguishes it from other methods that estimate harmonic modes from the structural information (either from the anatomical connectome [47] or surface mesh [48]).

We analysed the fMRI data of the DMT-induced brain state with HADES. This allowed us to test the anarchic brain or ‘Relaxed Beliefs Under Psychedelics’ (REBUS) model, as well as findings of enhanced signatures of criticality under these compounds [21,46,49]. We hypothesized that the DMT state is associated with a flatter spacetime hierarchy of cortical functional organization with enhanced integrative properties across the cortex.

## RESULTS

**Figure 1.**
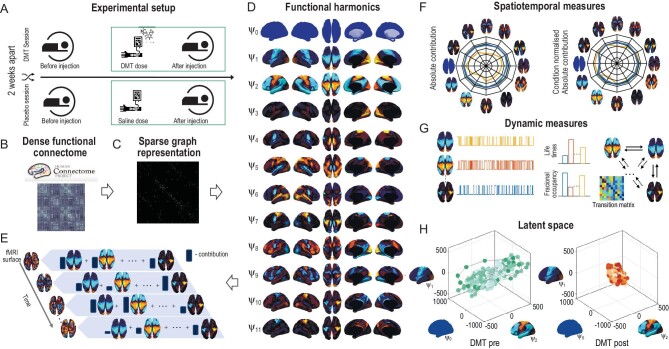
Overview of HArmonic DEcomposition of Spacetime (HADES) framework. (A) Here we used HADES to analyse data from DMT-induced resting-state fMRI in healthy participants and show the design for this experiment. (B) HADES uses the dense functional connectome constructed from the HCP S1200 release of 812 subjects to (C) construct a graph representation as a sparse, symmetric, and binary adjacency matrix of the dense functional connectome. (D) First, functional harmonics (${{\psi }_k}$(x)) are obtained from the Laplacian decomposition of the sparse adjacency matrix. (E) Functional harmonic decomposition is computed by projecting individual harmonics on the fMRI timeseries (surface representation) and calculating their contributions. (F) From this decomposition, HADES can be used to compute spatiotemporal measures for the first 11 FHs and 0th global FH—absolute contribution and condition normalized absolute contribution on any neuroimaging dataset. (G) Importantly, HADES can also be used to construct dynamic measures for the first 11 FHs and 0th global FH—fractional occupancy, life times and transition matrix. (H) These measures can be used as latent space representation in terms of temporal trajectory embedded in the functional harmonic space.

HADES describes the spatiotemporal dynamics in terms of spatial bases (defined from the brain's communication structure) and the spatial bases functional contributions to the fMRI recording evolving in time. To do so, we first constructed dense functional connectome from the Human Connectome Project (HCP) S1200 release of 812 subjects (Fig. 1B). The dense functional connectome was represented as a sparse, symmetric, and binary adjacency matrix (Fig. 1C) and decomposed into the functional harmonics (${{{\mathrm{\psi }}}_{\mathrm{k}}}$(x)) using the eigen-decomposition of the graph Laplacian applied to the dense functional connectome (Fig. 1D). Consistent with [4], we focused our analysis on the first 11 lowest functional harmonics together with the 0th global harmonic. We analysed functional significance of the functional harmonics by comparing them to the Yeo seven and 17th functional networks (Fig. S1). To obtain the temporal signature, we further projected the individual harmonics on the fMRI timeseries (in surface representation), using functional harmonic decomposition, and thus calculated the FHs temporal weights (Fig. 1E). We reconstructed the timeseries with a few harmonics to motivate the similarity to the empirical data (Fig. S2). Then, using a collection of spatiotemporal and dynamic measures (Fig. 1F and G) and latent space representation (Fig. 1H), we applied HADES to study the DMT state and its functional reorganization in terms of flattening of functional hierarchies and integrative properties across the cortex.

### Absolute contribution across functional harmonics

**Figure 2.**
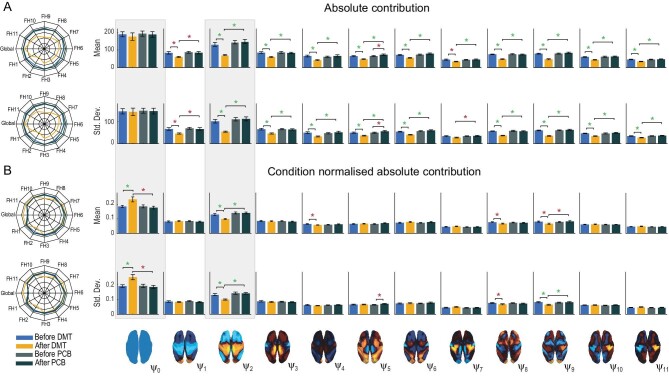
Spatiotemporal analysis of DMT and placebo neuroimaging data. The harmonic spatiotemporal analysis of the neuroimaging data shows that the contribution of functional harmonic ${{\psi }_2}$ (FH${{\psi }_2}$) is very significantly reduced (*P* < 0.05, Bonferroni corrected) when participants were given DMT, both in terms of absolute and normalized contribution. (A) Specifically, the absolute contribution across the first 11 FHs and the 0th global FH is shown both visually, on a spider plot, and statistically for individual FH across the four DMT-based conditions. The results show a decrease in the DMT-induced state (compared to DMT before injection and the placebo state) across many of the 11 FHs except the global FH ${\mathrm{\ }}{{\psi }_0}$ (green star: *P* < 0.05, Bonferroni corrected paired t-test; red star: *P* < 0.05, not Bonferroni corrected paired t-test). (B) Equally, we show the normalized absolute contribution across the first 11 FHs and the 0th global FH represented both visually, on a spider plot, and statistically for individual FHs across the four DMT-based conditions. Again, the results demonstrate an increase in the global FH ${\mathrm{\ }}{{\psi }_0}$ but specifically a decrease in FH ${{\psi }_2}$ compared to DMT before injection and the placebo state (green star: *P* < 0.05, Bonferroni corrected paired t-test; red star: *P* < 0.05 not, Bonferroni corrected paired t-test).

To quantify contributions of individual harmonics in the different conditions, we computed the absolute and condition-normalized absolute contributions of each harmonic (Fig. 2A). The absolute contribution results show a decrease in the DMT-induced state (compared to DMT before injection and placebo-induced states) across most of the 11 FHs except of the 0th global FH. This is contrasted by the condition-normalized absolute contribution results demonstrating an increase in the global FH and a decrease in FH 2 after DMT injection versus before injection and the placebo data (Fig. 2B). Spider plots in Fig. 2A and B represent a visual redistribution of FHs across different conditions for the two measures.

### Dynamic measures of HADES

**Figure 3.**
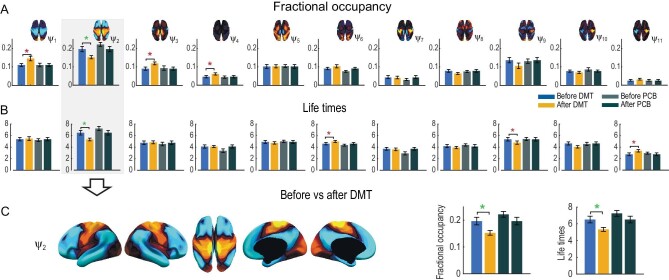
Dynamic analysis for the 11 FHs. Extending the spatial analysis into the spatiotemporal domain shows that functional harmonic ${{\psi }_2}$ (FH${{\psi }_2}$) is significantly reduced in the DMT condition. (A) Specifically, fractional occupancy was found to be statistically different in the ${{\psi }_2}$. (B) Lifetimes were found statistically different in the ${{\psi }_2}$ (green star: *P*-value < 0.05 (# of ${{\psi }_n}$), where *n* = 11, paired t-test; red star: *P*-value < 0.05, uncorrected paired t-test). (C) The full spatial extent of FH ${{\psi }_2}$ is shown along with the significant results for fractional occupancy and lifetimes.

To assess the temporal evolution of FH weights, we apply a winner-takes-all approach whereby we select the most prominent FH at every time point and compute fractional occupancy (FO) and lifetimes (LT) of each FH. In Fig. 3A and B, we show results when choosing the 11 FHs. We excluded the 0th FH in this analysis to focus on the dynamical properties of functionally resolved FHs. As before, strongest statistical significance for FO and LT is observed in ${{\psi }_2}$ (Fig. 3C). Furthermore, we computed the first order Markov process in terms of the Transition Probability Matrix (TPM) (Fig. S3A). We report statistics for the two DMT conditions (*P*-value < 0.05, uncorrected paired t-test).

### Latent space

**Figure 4.**
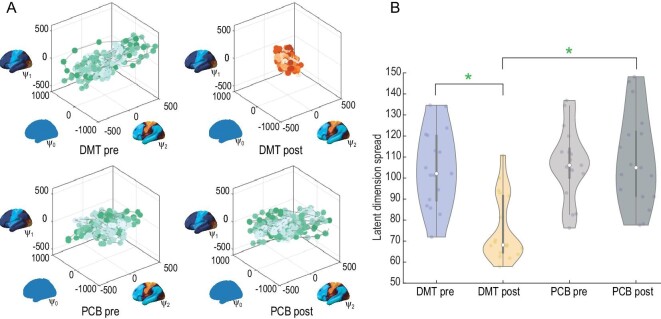
Latent space representation using the 11 FHs and the 0th global FH. Importantly, HADES can be used to create a latent space representation of the DMT neuroimaging data that immediately brings out important spacetime differences. (A) Here we show the figures with latent space representation using the first three FHs for visualization of the neuroimaging data. The green colour shading represents the temporal trajectory embedded in the three latent spatial dimensions of the FHs of DMT pre, PCB pre and PCB post. As can be immediately seen for the DMT-induced state (DMT post), there is a clear contraction of the contribution of the FHs across board (shown in red colour shading). (B) This can be directly quantified in terms of the latent dimension spread computed for all the 11 FHs and the 0th global FH i.e. 12th dimensional space for the four conditions. As can be seen, DMT post is significantly different from DMT pre and PCB post (green star: *P* < 0.05, Bonferroni corrected paired t-test).

Functional harmonics were used as the basis of a latent space representation in which the temporal trajectory of the brain dynamics was embedded in the latent space representation of the 11 FHs and the 0th global FH (Fig. 4A, here visualized for the first three FHs with colour shading representing the temporal trajectory). To further analyse how the temporal embedding in this latent space changes, we defined the expansion/contraction of the trajectory in terms of the latent dimension spread. The DMT-induced state contracts the contribution of the FHs across the board. Latent dimension spread was computed for all 11 FHs and the 0th global FH i.e. 12th dimensional space for the four conditions. We also report its statistics (green star *P*-value <0.05 Bonferroni corrected paired t-test). The temporal trajectory significantly contracts in the DMT-induced state.

## DISCUSSION

In this study, we analysed spacetime hierarchy of the DMT-induced brain state in healthy participants using the HADES framework. We found a significant change of brain spacetime hierarchy in line with the theoretical predictions of the REBUS theory [25] and the anarchic brain hypothesis, integrating Friston's free-energy principle [50] with Carhart-Harris’ entropic brain hypothesis [23,24].

Consistent with previous literature, we have demonstrated the functional relevance of functional harmonics [4]. Moreover, we have demonstrated that an empirical fMRI signal can be accurately reconstructed with a subset of functional harmonics. Applying HADES to the DMT-induced state has shown decreases in absolute contribution across most FHs, while the global FH has remained unchanged. However, when looking at condition-normalized absolute contribution in individual subjects, a decrease in FH ${{\psi }_2}$ was mirrored by an increase in the global harmonic. These results motivate a non-trivial reconfiguration whereby the DMT-induced state decreases in overall magnitude with a relative increase towards the global substate and a decrease of FH ${{\psi }_2}$ representative of the functional hierarchies of the brain. This was further reinforced by the analysis of functional harmonic dynamics with decreases both in fractional occupancy and lifetimes of FH ${{\psi }_2}$ demonstrating further dynamic collapse of this harmonic. Last, when the temporal trajectories were embedded in the latent space of the functional harmonic, the DMT-induced state showed significant contraction of its temporal trajectory spread.

Remarkably, FH ${{\psi }_2}\ $resembles the so-called ‘principal gradient’—i.e. a unimodal to transmodal gradient previously found to explain the greatest proportion of variance in a principal components analysis of cortical functional connectivity [3]. This gradient has been proposed to reflect a hierarchy of brain function from low- to high-order cognitive networks. This is in line with several theories of brain organization; namely REBUS and the anarchic brain where functional hierarchies are undermined under psychedelics [34,38,50], Temporo-spatial Theory of Consciousness where temporo-spatial nestedness becomes abnormal resulting in loss of spatial topographic organization [51], and the Operational Spacetime Theory where operational spacetime is hypothesized to be altered reflecting disruptions in the phenomenal spacetime [52]. Furthermore, the relative increase in global FH speaks to a less functionally defined and more integrated global substate under the influence of DMT. Indeed, at the RSN level, psychedelic-induced states have been shown to subvert within-functional network connectivity, especially in higher-order frontoparietal and default mode networks [11,22,53,54], while enhancing between-network connectivity and overall global and integrative tendencies [11,19].

Traditionally, neuroscience has focused on delineating and studying localized cortical regions to map the brain's function. Such an approach has been of importance albeit with fragmented insights as to how multiscale brain organization gives rise to complex spatiotemporal dynamics and ultimately behaviour. A recent development in system neuroscience has been that of cortical gradients [3]. This proposes an intrinsic coordinate system of human brain organization continuously varying from unimodal to transmodal cortical areas [31]. Gradient-type organization has been demonstrated in terms of myelination [55], anatomical structure [30], white matter tract length [56], evolutionary expansion [57], ontogenetic expansion [58], temporal processing [59], semantic processing [60] and physiologically coupled travelling waves [61]. The framework of multidimensional harmonic representation and decomposition [4,46,47] adds to this list by decomposing brain activity maps into frequency-specific communication channels that unveil contributions of connectivity gradients and cortical parcellations to brain function. HADES extends these frameworks by considering the dynamic aspects of these frequency-specific channels of functional communication.

The brain as a complex system has been hypothesized to manifest hierarchies across time and space. Indeed, such a nested organization was suggested both in terms of the structural architecture of the brain as well as its temporal frequencies [62,63]. Functional harmonics are by construction intrinsically ordered according to their spatial frequencies and as such provide a multiscale representation of brain activity across cortical space. Intuitively, spatial frequencies relate to temporal frequencies of oscillations where global spatial frequencies of harmonics reflect slow oscillations and local spatial frequencies of harmonics reflect fast oscillations. Drawing a closer relationship between the spatial and temporal scales is an important further step (possibly explore with M/EEG modalities) as the relevance of intrinsic neuronal timescales (INT) have been proposed for input sharing [64] with a hierarchical organization closely relating to the spatial organization of FH ${{\psi }_2}$ [57]. This hierarchical organization is important for temporal integration and segregation of input stimuli [65], with Default Mode Network possibly sitting at the apex integrating information over long timescales [66]. Indeed, recent work has shown how this temporal hierarchy changes in rest and task MEG data [67]. Yet how the changes in spatial distribution of the INT maps on the temporal frequencies of the functional harmonics remains to be seen. This could in part be due to the conceptual difference between the approaches whereby functional harmonics associate temporal frequency to individual harmonics, while INT mostly pertains to individual nodes.

Previously, connectome harmonics have been used to decompose the brain's spatiotemporal activity into a combination of time-varying contributions [46]. Using long-range and local connectivity as an underlying structure has been relevant in exploring the structure-function relationship of large-scale brain organization [47]. However, it seems that structural connectivity alone cannot explain the emergence of rich and spontaneous activity of the human brain [68,69]. First, neocortex is endowed with remarkable heterogeneity in cytoarchitecture. This will result in various computational differentiations across the cortex, for example in terms of temporal processing [59]. Second, the neuromodulatory system is known to alter the electrical composition of neurons and thus exercise non-linear effects on the emergent activity of various microcircuits across the brain [70,71]. The hypothesis here is that the communication structure of dense FC has implicitly embedded within it information on anatomical structure, cortical computational heterogeneity as well as neuromodulatory expression and as such serves as a prominent candidate to be used for the derivation of fundamental functional building blocks of spatiotemporal activity [4]. This in turn is expanded upon in the HADES framework with dynamic measures and latent space embeddings, whereby the emphasis is on the importance of the temporal dimension along which these spatiotemporal blocks building unfold.

Latent space representation has become an important research topic in neuroscience due to its ability to retrieve meaningful features contained in large and complex datasets [72]. It is possible to identify patterns and relationships in a lower-dimensional space between regions and between cognitive processes as the underlying computations giving rise to cognitive functions are likely to be integrated [1]. There are many techniques that serve this purpose from more traditional linear approaches such as singular value decomposition or principal component analysis [73], to popular techniques based on independent component analysis [74]. More recent works use autoencoders as an elegant way of compressing the fMRI signal while accounting for non-linearity in the data [75]. Here, we chose functional harmonics as they preserve nonlinear relationship between regions and have multiscale and interpretable representation of its latent dimensions [4,76]. However, it is to be noted that the idea of HADES as a framework goes beyond the actual representation of the dimension of the latent space (here in terms of functional harmonics) as it attempts to combine the spatial and temporal representation of complex brain dynamics. Moreover, in theory, other techniques could be applied in a similar way as to account for the complex spatiotemporal activity of the human brain.

A limitation of the current approach for describing functional harmonics propagating in time is that it might be too reductionist. ‘Winner-takes-all’ is a powerful technique summarizing the brain's dynamics in terms of fractional occupancy and lifetimes of the functional harmonics. However, it considers only one FH to be active at a given timepoint and as such might neglect other potential important information included in other FHs. Future work should implement weighted contributions of individual FHs at given timepoints and as such more completely describe the multidimensional representation of spatiotemporal dynamics. Another aspect for repeatability and robustness of the functional harmonics is the choice of the binarization of the adjacency matrix. Future work should for example investigate how the nearest-neighbours approach compares to distance-dependent binarizations [77] or data-driven topological approach [78].

## CONCLUSION

Taken all together, in this study we have examined the spatiotemporal dynamics of the brain under DMT with the sensitive and robust new HADES framework, which uses FHs derived from the brain's communication structure to model dynamics as weighted contributions of FHs evolving in time. Overall, we corroborate the REBUS and anarchic brain model of psychedelic action by demonstrating dynamic changes to brain's functional spacetime hierarchies.

## METHODS

Detailed methods and materials are given in the online supplementary data.

## CODE AVAILABILITY

Codes to apply the HADES framework and to follow the DMT analysis can be found publicly available at https://github.com/jvohryzek/HADES

## Supplementary Material

nwae124_Supplemental_File

## References

[1] Vohryzek J, Cabral J, Vuust P et al. Understanding brain states across spacetime informed by whole-brain modelling. Phil Trans R Soc A 2022; 380: 20210247. 10.1098/rsta.2021.0247 35599554 PMC9125224

[2] Felleman DJ, Van Essen DC. Distributed hierarchical processing in the primate cerebral cortex. Cereb Cortex 1991; 1: 1–47. 10.1093/cercor/1.1.1 1822724

[3] Margulies DS, Ghosh SS, Goulas A et al. Situating the default-mode network along a principal gradient of macroscale cortical organization. Proc Natl Acad Sci USA 2016; 113: 12574–9. 10.1073/pnas.1608282113 27791099 PMC5098630

[4] Glomb K, Kringelbach ML, Deco G et al. Functional harmonics reveal multi-dimensional basis functions underlying cortical organization. Cell Rep 2021; 36: 109554. 10.1016/j.celrep.2021.109554 34433059 PMC8411120

[5] Preti MG, Bolton TA, Van De Ville D. The dynamic functional connectome: state-of-the-art and perspectives. Neuroimage 2017; 160: 41–54. 10.1016/j.neuroimage.2016.12.061 28034766

[6] Northoff G, Wainio-Theberge S, Evers K. Is temporo-spatial dynamics the ‘common currency’ of brain and mind? In quest of ‘spatiotemporal neuroscience’. Phys Life Rev 2020; 33: 34–54. 10.1016/j.plrev.2019.05.002 31221604

[7] Tagliazucchi E . Time is a river which sweeps consciousness along, but consciousness is the river. Phys Life Rev 2020; 33: 75–7. 10.1016/j.plrev.2019.09.010 31624013

[8] Luppi AI, Vohryzek J, Kringelbach ML et al. Distributed harmonic patterns of structure-function dependence orchestrate human consciousness. Commun Biol 2023; 6: 117. 10.1038/s42003-023-04474-1 36709401 PMC9884288

[9] Nichols DE . Psychedelics. Pharmacol Rev 2016; 68: 264–355. 10.1124/pr.115.011478 26841800 PMC4813425

[10] Timmermann C, Roseman L, Schartner M et al. Neural correlates of the DMT experience assessed with multivariate EEG. Sci Rep 2019; 9: 16324. 10.1038/s41598-019-51974-4 31745107 PMC6864083

[11] Timmermann C, Roseman L, Haridas S et al. Human brain effects of DMT assessed via EEG-fMRI. Proc Natl Acad Sci USA 2023; 120: e2218949120. 10.1073/pnas.2218949120 36940333 PMC10068756

[12] Timmermann C, Roseman L, Williams L et al. DMT models the near-death experience. Front Psychol 2018; 9: 1424. 10.3389/fpsyg.2018.01424 30174629 PMC6107838

[13] Davis Z, Muller L, Trujillo JM et al. Spontaneous traveling cortical waves gate perception in awake behaving primates. Nature 2020; 587: 432–6. 10.1038/s41586-020-2802-y 33029013 PMC7677221

[14] SPL026 (DMT Fumarate) in Healthy Subjects and MDD Patients. ClinicalTrials.gov Identifier: NCT04673383 . https://clinicaltrials.gov/study/NCT04673383 (19 April 2024, date last accessed).

[15] Palhano-Fontes F, Barreto D, Onias H et al. Rapid antidepressant effects of the psychedelic ayahuasca in treatment-resistant depression: a randomized placebo-controlled trial. Psychol Med 2019; 49: 655–63. 10.1017/S0033291718001356 29903051 PMC6378413

[16] Carhart-Harris RL, Chandaria S, Erritzoe DE et al. Canalization and plasticity in psychopathology. Neuropharmacology 2023; 226: 109398. 10.1016/j.neuropharm.2022.109398 36584883

[17] Vohryzek J, Cabral J, Lord L et al. Brain dynamics predictive of response to psilocybin for treatment-resistant depression. bioRxiv: 2022.06.30.497950. 10.1093/braincomms/fcae049 PMC10957168 38515439

[18] Ruffini G, Lopez-Sola E, Vohryzek J et al. Neural geometrodynamics : a psychedelic perspective. bioRxiv: 2023.08.14. 553258. 10.3390/e26010090 PMC11154528 38275498

[19] Tagliazucchi E, Carhart-Harris R, Leech R et al. Enhanced repertoire of brain dynamical states during the psychedelic experience. Hum Brain Mapp 2014; 35: 5442–56. 10.1002/hbm.22562 24989126 PMC6869695

[20] Atasoy S, Deco G, Kringelbach ML et al. Harmonic brain modes: a unifying framework for linking space and time in brain dynamics. Neuroscientist 2018; 24: 277–93. 10.1177/1073858417728032 28863720

[21] Atasoy S, Vohryzek J, Deco G et al. Common neural signatures of psychedelics: frequency-specific energy changes and repertoire expansion revealed using connectome-harmonic decomposition. Prog Brain Res 2018; 242: 97–120. 10.1016/bs.pbr.2018.08.009 30471684

[22] Lord LD, Expert P, Atasoy S et al. Dynamical exploration of the repertoire of brain networks at rest is modulated by psilocybin. Neuroimage 2019; 199: 127–42. 10.1016/j.neuroimage.2019.05.060 31132450

[23] Carhart-Harris RL, Leech R, Hellyer PJ et al. The entropic brain: a theory of conscious states informed by neuroimaging research with psychedelic drugs. Front Hum Neurosci 2014; 8: 20. 10.3389/fnhum.2014.00020 24550805 PMC3909994

[24] Carhart-Harris RL . The entropic brain—revisited. Neuropharmacology 2018; 142: 167–78. 10.1016/j.neuropharm.2018.03.010 29548884

[25] Carhart-Harris RL, Friston KJ. REBUS and the anarchic Brain: toward a unified model of the Brain action of psychedelics. Pharmacol Rev 2019; 71: 316–44. 10.1124/pr.118.017160 31221820 PMC6588209

[26] Eickhoff SB, Yeo BTT, Genon S. Imaging-based parcellations of the human brain. Nat Rev Neurosci 2018; 19: 672–86. 10.1038/s41583-018-0071-7 30305712

[27] Eickhoff SB, Constable RT, Yeo BTT. Topographic organization of the cerebral cortex and brain cartography. Neuroimage 2018; 170: 332–47. 10.1016/j.neuroimage.2017.02.018 28219775 PMC5563483

[28] Cahalane DJ, Charvet CJ, Finlay BL. Modeling local and cross-species neuron number variations in the cerebral cortex as arising from a common mechanism. Proc Natl Acad Sci USA 2014; 111: 17642–7. 10.1073/pnas.1409271111 25422426 PMC4267349

[29] Charvet CJ, Cahalane DJ, Finlay BL. Systematic, cross-cortex variation in neuron numbers in rodents and primates. Cereb Cortex 2015; 25: 147–60. 10.1093/cercor/bht214 23960207 PMC4259279

[30] Burt JB, Demirtas M, Eckner WJ et al. Hierarchy of transcriptomic specialization across human cortex captured by structural neuroimaging topography. Nat Neurosci 2018; 21: 1251–9. 10.1038/s41593-018-0195-0 30082915 PMC6119093

[31] Huntenburg JM, Bazin PL, Margulies DS. Large-scale gradients in Human cortical organization. Trends Cogn Sci 2018; 22: 21–31. 10.1016/j.tics.2017.11.002 29203085

[32] Sereno MI, Pitzalis S, Martinez A. Mapping of contralateral space in retinotopic coordinates by a parietal cortical area in humans. Science 2001; 294: 1350–4. 10.1126/science.1063695 11701930

[33] Perrone-Capano C, Volpicelli F, Di Porzio U. Biological bases of human musicality. Rev Neurosci 2017; 28: 235–45. 10.1515/revneuro-2016-0046 28107174

[34] Kaufman IC . The cerebral cortex of man: a clinical study of localization of function. Am J Psychiatry 1951; 108: 153. 10.1176/ajp.108.2.153

[35] Haak KV, Marquand AF, Beckmann CF. Connectopic mapping with resting-state fMRI. Neuroimage 2018; 170: 83–94. 10.1016/j.neuroimage.2017.06.075 28666880

[36] Fox MD, Snyder AZ, Vincent JL et al. The human brain is intrinsically organized into dynamic, anticorrelated functional networks. Proc Natl Acad Sci USA 2005; 102: 9673–8. 10.1073/pnas.0504136102 15976020 PMC1157105

[37] Allen EA, Damaraju E, Plis SM et al. Tracking whole-brain connectivity dynamics in the resting state. Cereb Cortex 2014; 24: 663–76. 10.1093/cercor/bhs352 23146964 PMC3920766

[38] Tagliazucchi E, Balenzuela P, Fraiman D et al. Criticality in large-scale brain fMRI dynamics unveiled by a novel point process analysis. Front Physiol 2012; 3: 15. 10.3389/fphys.2012.00015 22347863 PMC3274757

[39] Karahanoğlu FI, Van De Ville D. Transient brain activity disentangles fMRI resting-state dynamics in terms of spatially and temporally overlapping networks. Nat Commun 2015; 6: 7751. 10.1038/ncomms8751 26178017 PMC4518303

[40] Griffa A, Ricaud B, Benzi K et al. Transient networks of spatio-temporal connectivity map communication pathways in brain functional systems. Neuroimage 2017; 155: 490–502. 10.1016/j.neuroimage.2017.04.015 28412440

[41] Vohryzek J, Griffa A, Mullier E et al. Dynamic spatio-temporal patterns of brain connectivity reorganize across development. Netw Neurosci 2020; 4: 115–33. 10.1162/netn_a_00111 32043046 PMC7006876

[42] Baker AP, Brookes MJ, Rezek IA et al. Fast transient networks in spontaneous human brain activity. eLife 2014; 3: e01867. 10.7554/eLife.01867 24668169 PMC3965210

[43] Vidaurre D, Smith SM, Woolrich MW. Brain network dynamics are hierarchically organized in time. Proc Natl Acad Sci USA 2017; 114: 12827–32. 10.1073/pnas.1705120114 29087305 PMC5715736

[44] Cabral J, Vidaurre D, Marques P et al. Cognitive performance in healthy older adults relates to spontaneous switching between states of functional connectivity during rest. Sci Rep 2017; 7: 5135. 10.1038/s41598-017-05425-7 28698644 PMC5506029

[45] Vohryzek J, Deco G, Cessac B et al. Ghost attractors in spontaneous brain activity: recurrent excursions into functionally-relevant BOLD phase-locking states. Front Syst Neurosci 2020; 14: 20. 10.3389/fnsys.2020.00020 32362815 PMC7182014

[46] Atasoy S, Roseman L, Kaelen M et al. Connectome-harmonic decomposition of human brain activity reveals dynamical repertoire re-organization under LSD. Sci Rep 2017; 7: 17661. 10.1038/s41598-017-17546-0 29247209 PMC5732294

[47] Atasoy S, Donnelly I, Pearson J. Human brain networks function in connectome-specific harmonic waves. Nat Commun 2016; 7: 10340. 10.1038/ncomms10340 26792267 PMC4735826

[48] Pang JC, Aquino KM, Oldehinkel M et al. Geometric constraints on human brain function. Nature 2023; 618: 566–74. 10.1038/s41586-023-06098-1 37258669 PMC10266981

[49] Toker D, Pappas I, Lendner JD et al. Consciousness is supported by near-critical slow cortical electrodynamics. Proc Natl Acad Sci USA 2022; 119: e2024455119. 10.1073/pnas.2024455119 35145021 PMC8851554

[50] Friston K, Kilner J, Harrison L. A free energy principle for the brain. J Physiol Paris 2006; 100: 70–87. 10.1016/j.jphysparis.2006.10.001 17097864

[51] Northoff G, Zilio F. Temporo-spatial Theory of Consciousness (TTC)—Bridging the gap of neuronal activity and phenomenal states. Behav Brain Res 2022; 424: 113788. 10.1016/j.bbr.2022.113788 35149122

[52] Fingelkurts AA, Fingelkurts AA, Neves CFH. Natural world physical, brain operational, and mind phenomenal space-time. Phys Life Rev 2010; 7: 195–249. 10.1016/j.plrev.2010.04.001 20417160

[53] Carhart-Harris RL, Muthukumaraswamy S, Roseman L et al. Neural correlates of the LSD experience revealed by multimodal neuroimaging. Proc Natl Acad Sci USA 2016; 113: 4853–8. 10.1073/pnas.1518377113 27071089 PMC4855588

[54] Palhano-Fontes F, Andrade KC, Tofoli LF et al. The psychedelic state induced by ayahuasca modulates the activity and connectivity of the default mode network. PLoS One 2015; 10: e0118143. 10.1371/journal.pone.0118143 25693169 PMC4334486

[55] Huntenburg JM, Bazin PL, Goulas A et al. A systematic relationship between functional connectivity and intracortical myelin in the Human cerebral cortex. Cereb Cortex 2017; 27: 981–97. 10.1093/cercor/bhx030 28184415 PMC5390400

[56] Buckner RL, Krienen FM. The evolution of distributed association networks in the human brain. Trends Cogn Sci 2013; 17: 648–65. 10.1016/j.tics.2013.09.017 24210963

[57] Rilling JK . Comparative primate neuroimaging: insights into human brain evolution. Trends Cogn Sci 2014; 18: 46–55. 10.1016/j.tics.2013.09.013 24501779

[58] Hill J, Inder T, Neil J et al. Similar patterns of cortical expansion during human development and evolution. Proc Natl Acad Sci USA 2010; 107: 13135–40. 10.1073/pnas.1001229107 20624964 PMC2919958

[59] Baldassano C, Chen J, Zadbood A et al. Discovering event structure in continuous narrative perception and memory. Neuron 2017; 95: 709–21. 10.1016/j.neuron.2017.06.041 28772125 PMC5558154

[60] Huth AG, Lee T, Nishimoto S et al. Decoding the semantic content of natural movies from human brain activity. Front Syst Neurosci 2016; 10: 81. 10.3389/fnsys.2016.00081 27781035 PMC5057448

[61] Raut RV, Snyder AZ, Mitra A et al. Global waves synchronize the brain's functional systems with fluctuating arousal. Sci Adv 2021; 7: eabf2709. 10.1126/sciadv.abf2709 34290088 PMC8294763

[62] Betzel RF, Bassett DS. Multi-scale brain networks. Neuroimage 2017; 160: 73–83. 10.1016/j.neuroimage.2016.11.006 27845257 PMC5695236

[63] He BJ . Scale-free brain activity: past, present, and future. Trends Cogn Sci 2014; 18: 480–7. 10.1016/j.tics.2014.04.003 24788139 PMC4149861

[64] Golesorkhi M, Gomez-Pilar J, Zilio F et al. The brain and its time: intrinsic neural timescales are key for input processing. Commun Biol 2021; 4: 970. 10.1038/s42003-021-02483-6 34400800 PMC8368044

[65] Wolff A, Berberian N, Golesorkhi M et al. Intrinsic neural timescales: temporal integration and segregation. Trends Cogn Sci 2022; 26: 159–73. 10.1016/j.tics.2021.11.007 34991988

[66] Yeshurun Y, Nguyen M, Hasson U. The default mode network: where the idiosyncratic self meets the shared social world. Nat Rev Neurosci 2021; 22: 181–92. 10.1038/s41583-020-00420-w 33483717 PMC7959111

[67] Golesorkhi M, Gomez-Pilar J, Tumati S et al. Temporal hierarchy of intrinsic neural timescales converges with spatial core-periphery organization. Commun Biol 2021; 4: 277. 10.1038/s42003-021-01785-z 33664456 PMC7933253

[68] Hagmann P, Cammoun L, Gigandet X et al. Mapping the structural core of human cerebral cortex. PLoS Biol 2008; 6: 1479–93. 10.1371/journal.pbio.0060159 PMC2443193 18597554

[69] Deco G, Jirsa VK. Ongoing cortical activity at rest: criticality, multistability, and ghost attractors. J Neurosci 2012; 32: 3366–75. 10.1523/JNEUROSCI.2523-11.2012 22399758 PMC6621046

[70] Shine JM . Neuromodulatory influences on integration and segregation in the brain. Trends Cogn Sci 2019; 23: 572–83. 10.1016/j.tics.2019.04.002 31076192

[71] Kringelbach ML, Cruzat J, Cabral J et al. Dynamic coupling of whole-brain neuronal and neurotransmitter systems. Proc Natl Acad Sci USA 2020; 117: 9566–76. 10.1073/pnas.1921475117 32284420 PMC7196827

[72] Cunningham JP, Yu BM. Dimensionality reduction for large-scale neural recordings. Nat Neurosci 2014; 17: 1500–9. 10.1038/nn.3776 25151264 PMC4433019

[73] Viviani R, Grön G, Spitzer M. Functional principal component analysis of fMRI data. Hum Brain Mapp 2005; 24: 109–29. 10.1002/hbm.20074 15468155 PMC6871761

[74] Smith SM, Miller KL, Moeller S et al. Temporally-independent functional modes of spontaneous brain activity. Proc Natly Acad Sci USA 2012; 109: 3131–6. 10.1073/pnas.1121329109 PMC3286957 22323591

[75] Perl YS, Boccacio H, Pérez-Ipiña I et al. Generative embeddings of brain collective dynamics using variational autoencoders. Phys Rev Lett 2020; 125: 238101. 10.1103/PhysRevLett.125.238101 33337222

[76] Vos de Wael R, Benkarim O, Paquola C et al. BrainSpace: a toolbox for the analysis of macroscale gradients in neuroimaging and connectomics datasets. Commun Biol 2020; 3: 103. 10.1038/s42003-020-0794-7 32139786 PMC7058611

[77] Belkin M, Niyogi P. Laplacian eigenmaps for dimensionality reduction and data representation. Neural Comput 2003; 15: 1373–96. 10.1162/089976603321780317

[78] Dimitriadis SI, Antonakakis M, Simos P et al. Data-driven topological filtering based on orthogonal minimal spanning trees: application to multigroup magnetoencephalography resting-State connectivity. Brain Connect 2017; 7: 661–70. 10.1089/brain.2017.0512 28891322 PMC6435350

