# Supplementary material for: The flattening of spacetime hierarchy of the N,N-dimethyltryptamine brain state is characterized by harmonic decomposition of spacetime (HADES) framework
Source: Natl Sci Rev. 2024 Apr 4;11(5):nwae124. doi: 10.1093/nsr/nwae124 (PMC11110867; doi:10.1093/nsr/nwae124)
Supplement: nwae124_Supplemental_File [file nwae124_supplemental_file.docx]

**Supplementary File**

**Harmonic decomposition of spacetime (HADES) framework characterises the spacetime hierarchy of the DMT brain state**

Jakub Vohryzek^1,2,3,4^, Joana Cabral^1,5,6^, Christopher Timmerman^7^, Selen Atasoy^1,2^, Leor Roseman^7^, David J Nutt^7^, Robin L Carhart-Harris^7,8^, Gustavo Deco^4,9,10,11^, Morten L Kringelbach^1,2,3^

1. Centre for Eudaimonia and Human Flourishing, Linacre College, University of Oxford
2. Department of Psychiatry, University of Oxford, Oxford, United Kingdom
3. Center for Music in the Brain, Aarhus University, Aarhus, Denmark
4. Center for Brain and Cognition, Computational Neuroscience Group, Department of Information and Communication Technologies, Universitat Pompeu Fabra, Barcelona, Spain.
5. Life and Health Sciences Research Institute, School of Medicine, University of Minho
6. ICVS/3B’s - PT Government Associate Laboratory, Braga/Guimarães, Portugal
7. Centre for Psychedelic Research, Department of Brain Sciences, Imperial College London, London, United Kingdom
8. Departments of Neurology and Psychiatry, University of California San Francisco, US
9. Institució Catalana de la Recerca i Estudis Avançats (ICREA), Barcelona, Spain
10. Department of Neuropsychology, Max Planck Institute for Human Cognitive and Brain Sciences, Leipzig, Germany
11. School of Psychological Sciences, Monash University, Melbourne, Australia

Corresponding author: Jakub Vohryzek, [jakub.vohryzek@upf.edu](mailto:jakub.vohryzek@upf.edu)

**Parcellation and Reference Functional Networks**

Functional Harmonics in vertex dimensions ($n = 59, 412$) were reduced to the Schaefer multiscale atlas of varying numbers of brain regions. Schaefer400 was applied in the main text to compare FH with the known canonical networks (7 and 17 Yeo resting-state networks [1]) but other scales were also considered (Schaefer100, 200, 300, 500 and 1000). The parcellated data was obtained by averaging vertices belonging to a given brain region as defined by the Schaefer parcellation. Unlike standard volumetric fMRI to atlas registration, the parcellated data were obtained in the surface space of 59, 412 vertices. I used the following Schaefer HCP surface templates [www.github.com/ThomasYeoLab](http://www.github.com/ThomasYeoLab).

**Functional Relevance**

Firstly, we compute and verify the overlap of Functional Harmonics with the canonical networks - specifically the 7 and 17 resting-state networks as described in the following study [1]. Previously, Glomb et al. have demonstrated the neurophysiological relevance of Functional Harmonics [2]. In **Figure SI 1A**, we display Functional Harmonics as computed both by HADES and by [2] for visual inspection. Apart from the Functional Harmonic 10, the patterns are consistent across the two datasets. As the calculation steps were kept identical, a possible discrepancy might have arisen from the dense functional connectome itself where an improved reconstruction software was used for the HADES dataset (in HADES: S1200 release of HCP subjects, in [2] S900 release of HCP subjects). Strikingly, the first two Functional Harmonics 1 and 2 reflect the principal gradients of human cortex delineating unimodal and transmodal networks of the brain [3] both in the 7 and 17 RSN implementation [1]. In the 17 RSN implementation, FHs 3 and 4 demonstrate separation of SMN and VIS. Moreover, FH5 places in opposite polarities the SMN and DMN (mainly its temporal parietal part) networks on one side, and DAN and FPN on the other. FH6 separates SMN and the temporal parietal part of the DMN from the rest of the DMN and FPN. FH 7 further differentiates the VIS and SM. FH 8 splits attention networks (mainly VAN) and DMN. FH 9 demonstrates gradient like separation between DMN and other higher-order cognitive networks (DAN, VAN, LN and FPN). FHs 10 and 11 strongly represent part of the FPN (and LN in the 7 RSNs representation) (**Figure SI 1B**).

*
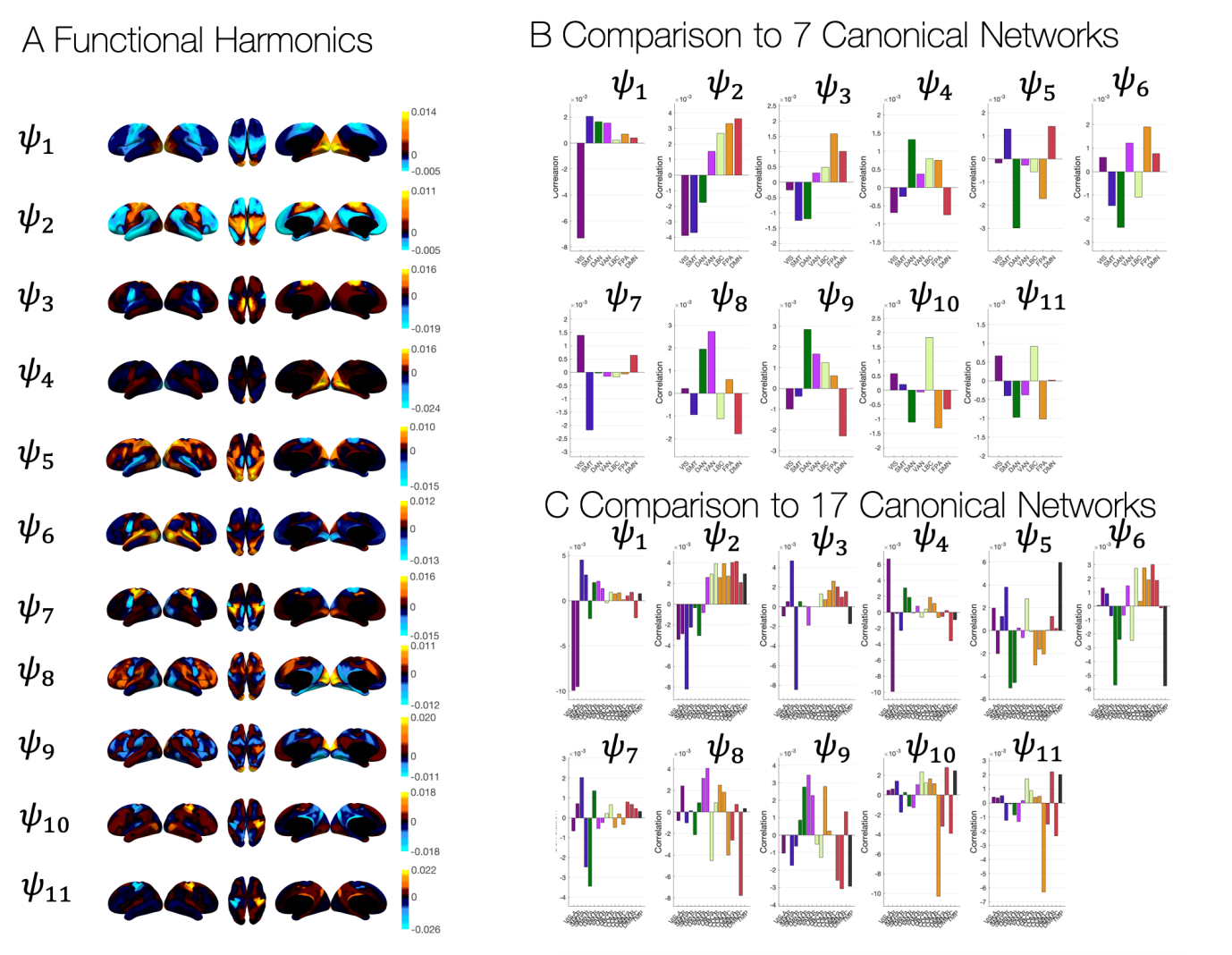
*

***Figure SI 1: Functional Harmonics:*** ***A)*** *The first 11 Functional Harmonics as obtained from the Laplacian eigendecomposition of the dense functional connectome.* ***B)*** *Comparison with 7 canonical resting-state networks.* ***C)*** *Comparison with 17 canonical resting-state networks* [1]*. Different Functional Harmonics indicate separation into various weighted combinations of resting state networks.*

**Functional Harmonic Reconstruction**

The reconstructed signal can be further defined as follows

$$\mathcal{F}^{R}\left( x, t_{i} \right)= \sum_{k=1}^{n} \psi_{k}\left( x \right)\tau_{k}(t_{i})$$

where $\mathcal{F}^{R}\left( x,t_{i} \right)$ is the reconstructed signal obtained from spatial components (Functional Harmonics), $\psi_{k}\left( x \right)$, and temporal components (contributions of Functional Harmonics), $\tau_{k}(t_{i})$, at every timepoint $t_{i}$ (**Figure 1 E**).

**Signal Reconstruction**

By projecting Functional Harmonics onto the timeseries, it is possible to obtain the contribution of each FH evolving in time. Henceforth, the underlying spatio-temporal activity is described in terms of its spatial (FH) and temporal (signal of FH contributions) dimensions and can be reconstructed back by linear summation (**Figure SI 2A**). For a template (DMT pre-treatment) condition, we show that reconstruction with 100 FHs achieves a correlation of 0.5, and 0.6 when the 0^th^ global FH is considered, with the original signal. Furthermore, when taking only the first 11 FHs for the reconstruction, the reconstructed signal correlates 0.4 with the original signal and further 0.5 when the 0th global FH is considered (**Figure SI 2B**).

*
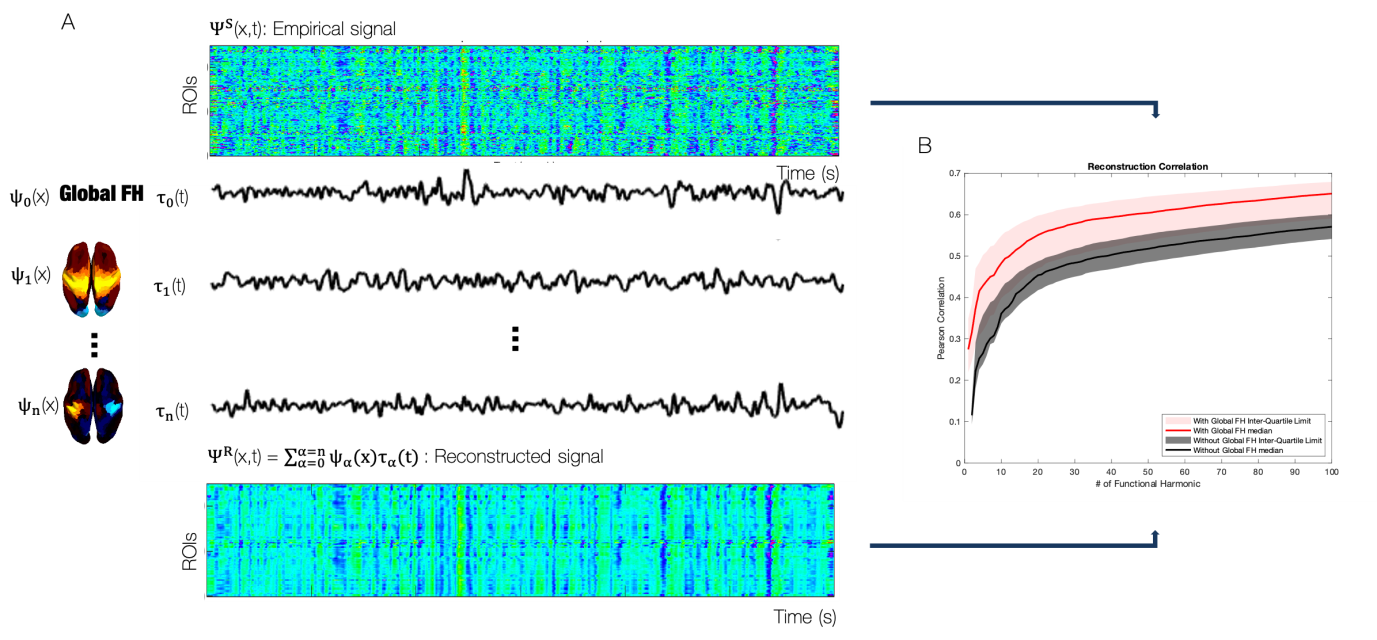
*

***Figure SI 2: Signal Reconstruction:*** *A) After the Functional Harmonic Decomposition, a signal can be reconstructed back by combining the spatial component (FHs) and temporal component (FH contribution). Furthermore, a good portion of the signal can be reconstructed with only a fraction of the FHs. B) Correlation of reconstruction between the empirical signal of DMT before injection and its reconstructed counterpart with varying number of FHs. The reconstruction with 100 FHs achieves a correlation of 0.5, and 0.6 with the 0^th^ global FH. Furthermore, only when considering the first 11 FHs, they correlate 0.4 and when the 0th global FH is included 0.5.*

**

***Figure SI 3: Transition Probability Matrix****. Transition Probability matrices for the two DMT conditions. We also report its statistics (p-value < 0.05 uncorrected paired t-test).*

[1] B. T. T. Yeo *et al.*, “The organization of the human cerebral cortex estimated by intrinsic functional connectivity.,” *J. Neurophysiol.*, vol. 106, no. 3, pp. 1125–65, Sep. 2011.

[2] K. Glomb, M. L. Kringelbach, G. Deco, P. Hagmann, J. Pearson, and S. Atasoy, “Functional harmonics reveal multi-dimensional basis functions underlying cortical organization,” *Cell Rep.*, vol. 36, no. 8, 2021.

[3] D. S. Margulies *et al.*, “Situating the default-mode network along a principal gradient of macroscale cortical organization,” *Proc. Natl. Acad. Sci. U. S. A.*, vol. 113, no. 44, pp. 12574–12579, Nov. 2016.

# Material and methods

## Experimental Data

## HCP Functional MRI

The dataset used for the analysis was made publicly available by the Human Connectome Project (HCP), WU-Minn Consortium (Principal Investigators: David Van Essen and Kamil Ugurbil: 1U54MH091657). This project was made possible by funding from the sixteen NIH Institutes and Centres supporting the NIH Blueprint for Neuroscience Research; and by the McDonell Centre for Systems Neuroscience at Washington University.

## Dense Functional Connectome

To define the appropriate functional basis, we used the dense functional connectome as part of the HCP 1200 Subject Release. The data is freely downloadable (with a connectomeDB account) at [https://db.humanconnectome.org](https://db.humanconnectome.org/data/projects/HCP_1200) under the zip-file called 812 Subjects, recon r227, Dense Connectome. Details about the dense functional connectome pipeline can be found on the same website under the following pdf ‘HCP1200- DenseConnectome +PTN+Appendix-July2017.pdf’. In brief, out of the 1200 HCP subjects, 1003 have undergone four rsfMRI runs (total of 4800 timepoints). An improved reconstruction software (’recon2’) was used on a further subset of 812 participants. Timeseries were minimally processed, had artefacts removed with ICA+FIX and were inter-subject registered. Further group-PCA was performed on the temporally demeaned and variance normalised timeseries. The outputs of the group-PCA are used to create the dense connectome. This can be thought of as a low-noise regularised equivalent of concatenating individual subject’s grey-ordinate timeseries and calculating the correlation between all the individual grey-ordinate timeseries, to create a dense functional connectome (**Figure 1A**).

## DMT dataset

The complete description of the participants, experimental design and acquisitions parameters can be found in (10,11). A group of 25 participants was recruited in a single-blind, placebo-controlled, and counter-balanced design. Subjects were considered for the study unless they were younger than 18 years of age, lacked experience with a psychedelic, had a previous negative response to a psychedelic and/or currently suffered from or had a history of psychiatric or physical illness. Out of the 25 participants, 20 completed the whole study (7 female, mean age = 33.5 years, SD = 7.9). A further 3 subjects were excluded due to excessive motion during the 8 minutes DMT recording (more than 15% of volumes scrubbed with framewise displacement (FD) of 0.4 mm).

## Experimental Paradigm

In total, all subjects were scanned on two days, two weeks apart, each consisting of two scanning sessions. The initial scan lasted 28 minutes with the 8th minute marking the intravenous administration of either DMT or placebo (saline) (50/50 DMT/placebo). Subjects were asked to lay in the scanner with their eyes closed (wearing an eye-mask). After the recording, assessment of subjective effects was carried out. The second session was identical to the first except for the assessment of subjective intensity scores at every minute of the recording. The experimental design also included simultaneous EEG recording during the sessions (**Figure 1A**).

## Acquisition Parameters

The experiment was performed on a 3T scanner (Siemens Magnetom Verio syngo MR 12) with compatibility for EEG recording. A T2 -weighted echo planar sequence was used. In brief, the parameters were as follows: TR/TE = 2000ms/30ms, acquisition time = 28.06 minutes, flip angle = 80o, voxel size = 3x3x3 mm^3^ and 35 slices with 0 mm interslice distance. T1-weighted structural scans of the brain were also acquired.

## fMRI Pre-processing

For fMRI pre-processing, a pipeline previously developed for an LSD experiment was used, which can be accessed in the supplementary information of (53). Briefly, the following steps were applied 1) despiking, 2) slice-timing correction, 3) motion correction, 4) brain extraction, 5) rigid body registration to structural scans, 6) non-linear registration to 2mm MNI brain, 7) motion-correction scrubbing, 8) spatial-smoothing (FWHM) of 6 mm, 9) bandpass filtering into the frequency range 0.01-0.08 Hz, 10) linear and quadratic detrending, 11) regression of 9 nuisance regressors (3 translations, 3 rotations and 3 anatomical signals). Lastly, the timeseries were projected from MNI voxel-space to the HCP surface vertex-space using the HCP command -volume-to-surface-mapping.

## Functional Harmonics

Functional harmonics are described by the eigenvectors of the Laplacian applied to a graph representation of the human brain’s communication structure (4). This graph is constructed as a binarization of the dense functional connectome $\mathfrak{R=(}\nu,\varepsilon)$, where each node, $\nu=\{x_{i}|\in1, \ldots, n\}$, corresponds to one of the n = 59 412 brain vertices and, for each node/vertex n, an edge, $\varepsilon=\{e_{ij}|\in\nu\times\nu\}$, is defined to the 300 most correlated vertices, according to the correlation values from the original dense functional connectome (**Figure 1B)**. The choice of 300 nearest neighbours was used following previous work by Glomb et al. showing that the resulting brain patterns (functional harmonics) preserve both global and local features of fMRI data [(4)](https://paperpile.com/c/5a5S5b/ZZ5o). Then, the resulting graph is thus a sparse, symmetric, and binary adjacency matrix (**Figure 1C**) as follows,

$$A\left( i,j \right)= \left\{ \begin{aligned} 1, if \left( i,j \right)\in\varepsilon\\ 0, otherwise \end{aligned} \right.$$

Then, the discrete counterpart of the Laplace operator, $\Delta,$ is applied to the adjacency matrix A in the following manner,

$$\Delta_{A} =D^{-1/2}L D^{-1/2}, with L=D-A$$

where D is the diagonal degree matrix, $D= \sum_{i=1}^{n} A(i,j)$. Lastly, Functional Harmonics, $\psi_{k}\left( x_{i} \right), k \in1, \ldots, n$ were computed as eigenvectors of the following eigenvalue problem,

$$\Delta_{A}\psi_{k}\left( x_{i} \right)= \lambda\psi_{k}\left( x_{i} \right),\forall x_{i} \in\nu$$

where $\lambda_{k}, k \in1, \ldots, n$ are the associated eigenvalues of $\Delta_{A}$ (**Figure 1D**).

## Functional Harmonic Decomposition

To describe how Functional Harmonics evolve in time, we weighted their contribution, $\tau$, for each participant at every timepoint, $t$, of the recording $\mathcal{F}^{s}(x,t)$, and thus, retrieved timecourses of individual harmonic contributions (**Figure 1D**) in the following format,

$$\mathcal{F}^{s}\left( x,t_{i} \right)= \sum_{k=1}^{n} \tau_{k}\left( t_{i} \right)\psi_{k}\left( x \right)= \tau_{1}\left( t_{i} \right)\psi_{1}\left( x \right)+ \tau_{2}\left( t_{i} \right)\psi_{2}\left( x \right)+\ldots+ \tau_{n}\left( t_{i} \right)\psi_{n}\left( x \right)$$

where $\tau_{k}$ is the contribution of the $k^{th}$ Functional Harmonic $\psi_{k}\left( x \right)$ to the fMRI recording $\mathcal{F}^{s}\left( x,t_{i} \right)$ at time $t_{i}$. Formally, the Functional Harmonic contributions are described as $\tau_{k}\left( t \right)= \left\langle\mathcal{F}^{s}\left( x,t \right),\psi_{k} \right\rangle$ (**Figure 1E**). In this light, the projection timecourses of functional harmonics, derived from the HCP group-based static dense functional connectome, can be seen as a deviation of the time snapshots of brain activity from the overall static dense functional connectome.

**Choosing the number of Functional Harmonics**

In previous work Glomb et al. have shown the global and local functional relevance of the first 11 functional harmonics both in resting-state and task data as well as its superiority to other dimensionality reduction techniques [(2)](https://paperpile.com/c/5a5S5b/pdCQ). We have therefore focused our analysis on the previously validated functional harmonics. Furthermore, a recent analysis has shown that in large-scale fMRI data (whether 100, 200 or 1000 parcels), there is an optimal dimension of latent representation which converges on roughly 10 dimensions [(4)](https://paperpile.com/c/5a5S5b/ZZ5o). This is indeed consistent with our choice of the number of functional harmonics. This point can be further appreciated in the reconstruction plot (see Figure SI 2) where the elbow of reconstruction is steepest for the first few functional harmonics suggesting the disproportionately significant contributions of these harmonics to the overall timeseries reconstruction.

## Spatiotemporal Measures

Functional Harmonic contribution $\tau_{k}(t)$ at each timepoint $t$ represents the weight of a given Functional Harmonic $\psi_{k}\left( x \right)$ at that fMRI timepoint, $\mathcal{F}^{R}\left( x,t_{i} \right)$. Its absolute value can be defined as the absolute contribution as follows: $P\left( \psi\left( x \right),t \right)= \left| \tau_{k}(t) \right|$. Here, we further define the mean absolute and condition-normalised absolute contribution as the time-averaged overall absolute contribution of each harmonic, and as the time-averaged condition-normalised absolute contribution by the sum of all the Functional Harmonic magnitudes of each participant and condition, respectively. In other words, absolute contribution describes the overall state of each Functional Harmonic for every participant and condition, and condition-normalised absolute contribution depicts the relative redistribution for a given Functional Harmonic in relationship to the rest of the Functional Harmonics (**Figure 1F**).

## Dynamic Measures

To summarise dynamics of Functional Harmonics, we chose to describe each timepoint by its dominant Functional Harmonics, i.e., a Functional Harmonic with the largest contribution at a given timepoint. As such, we were able to depict the individual timeseries as a sequence of dominant Functional Harmonic contributions. We further defined Fractional Occupancy, Life Times and Transition matrix.

**Fractional Occupancy**

The probabilities - or fractional occupancies - $\Pi_{\psi}^{(S)}$ belonging to each FH $\psi$ and each scan *S*, is derived as follows:

$$\Pi_{\psi}^{(S)}= \frac{1}{T}\sum_{t=1}^{T} \chi\left[ \underline{x}\left( t \right) \epsilon R^{\psi} \right] (1)$$

with $\chi$ is the indicator function - $\chi\left( A \right)=1$ if A is true, and $\chi\left( A \right)=0$ otherwise, and T=240 is the number of time points (TRs) corresponding to each fMRI scan *(S)*. Essentially, the equation tallies the occurrences where the $\underline{x}\left( t \right)$ falls within each of the defined FH $R^{(\psi)}$, then divides by the total number of time points T. Additionally, assuming participants remain in a resting state, meaning they aren't engaged in any tasks, we presume data stationarity within each scan, thus justifying the time average in equation 1. FH probabilities are estimated independently for each individual fMRI scan.

**Life Times**

To characterize the average duration of visits to a specific FH $\psi$ within each fMRI scan S, the dwell time ${LT}_{\psi}^{(S)}$ is defined as the average length of all consecutive periods spent in that FH. In other words,

$${LT}_{\psi}^{(S)}= \frac{1}{p_{\psi}}\sum_{1}^{p_{\psi}} C_{p_{\psi}} (2)$$

with ${LT}_{\alpha}$ being the Life Times of FH $\psi$, $p_{\psi}$ is the total number of consecutive periods assigned to specific FH $\psi$ and $C_{p_{\psi}}$is the duration of each consecutive period.

**Markov Chain Transition Probabilities**

Using the same rational as in equation (1), we can express the probability $\Pi_{\psi_{i}\psi_{j}}$ of FH $\psi$ at time **bin** $t$ and in FH $\psi$ at time **bin** $t+1$ as following:

$$\Pi_{\psi_{i}\psi_{j}}^{(S)}= \frac{1}{T-1} \sum_{t=1}^{T-1} \chi\left[ \underline{x}\left( t \right) \epsilon R^{\psi_{i}},\underline{x}\left( t +1 \right) \epsilon R^{\psi_{j}} \right]. (3)$$

As a result, the transition probability matrix $W_{\psi_{i}\psi_{j}}^{(S)}$ of every fMRI scan *S* comes out as:

$$W_{\psi_{i}\psi_{j}}^{(S)}=P\left[ \underline{x}\left( t+1 \right) \epsilon R^{\psi_{j}} | \underline{x}\left( t \right) \epsilon R^{\psi_{i}} \right]= \frac{\Pi_{\psi_{i}\psi_{j}}^{(S)}}{\Pi_{\psi_{i}}^{(S)}} . (4)$$

The matrix $W_{\psi_{i}\psi_{j}}$delineates the transition from FH $\psi_{i}$to FH $\psi_{j}$, establishing a homogeneous Markov chain that characterizes the shifts between FHs. The transition probability matrix $W_{\psi_{i}\psi_{j}}^{(S)}$ is computed independently for each scan S. Each $\boldsymbol{W}_{\psi_{i}\psi_{j}}^{\boldsymbol{(S)}}$ matrix corresponds to a transition graph, with an arrow directed from FH $\psi_{i}$to FH $\psi_{j}$if the value in the matrix is greater than 0. We opt to illustrate the transition graph by averaging $W_{\psi_{i}\psi_{j}}$across all subjects in both the DMT pre and DMT post conditions (see **Figure 1G and Figure SI 3**).

## Latent Space

Latent space serves as a lower-dimensional representation of high-dimensional data. Here, we have used the spatial patterns, described by Functional Harmonics, to embed the temporal activity in N-dimensional space where N is the number of FHs. As such it is possible to quantify the changes in temporal dynamics of FHs. Here, we define measure of Latent Dimension Spread that quantifies the amount of temporal trajectory expansion or contraction. It is defined as the average of the 11 FHs and the 0^th^ Global FH of the standard deviation of the Functional Harmonic contribution $\tau_{k}(t)$ over time (**Figure 1H**).
